# Revised Lifetimes of Energy Levels in Neutral Iron

**DOI:** 10.6028/jres.080A.072

**Published:** 1976-10-01

**Authors:** C. H. Corliss, J. L. Tech

**Affiliations:** Institute for Basic Standards, National Bureau of Standards, Washington, D.C. 20234

**Keywords:** Atomic spectra, energy levels, Fe I, iron, iron lifetimes, lifetimes in Fe I

## Abstract

Mean radiative lifetimes for 408 energy levels of neutral iron are revised from our 1967 paper on the basis of comparison with 81 subsequently measured lifetimes. The standard deviation of the ratio of the revised values to the reference lifetimes is 30 percent.

In 1967 the present authors published a comprehensive set of mean radiative lifetimes for 408 energy levels of neutral iron. The lifetimes were calculated by summing the transition probabilities for most of the important downward transitions from the levels. The transition probabilities used in the calculations were taken from a compilation by Corliss and Tech [1968] 1 that has since been shown by independent investigations at the University of Kiel, Harvard College Observatory, the California Institute of Technology, and other laboratories [see, for example, Bridges and Kornblith, 1974] to suffer from a systematic error. This systematic error in the transition probabilities, which propagated also into the calculation of lifetimes, resulted from a temperature-related error that gave an incorrect correlation between the energy level value and the level population, the higher levels being calculated as under populated.

Despite this known systematic error, our previous compilation continues to be cited and remains the largest collection of available lifetimes. It may be useful, therefore, to present now a revised compilation that corrects this systematic error on the basis of the most accurate independent direct measurements available. Since 1968 there have been ten publications from eight different laboratories reporting lifetimes for 57 levels in neutral iron that are based on 85 new direct measurements. By comparing these new results with our earlier values, we find that the correction to be made to our values is a quite well defined one, making it possible to improve considerably the values in the previous compilation and to bring them onto the scale of the best new measurements.

In figure 1, the logarithm of the ratio of the new measurements to our previous values is plotted against the energy value of the level. The digits appearing on this plot indicate the position and, in the case of near coincidences, the number of points involved. Four of the 85 ratios available are not plotted here because they are widely discrepant. The straight line on this plot represents the least squares linear fit to the remaining 81 points. The standard deviation of the points from the line is 30 percent. The equation of this line, which has been used to calculate the revised values of lifetimes for the 408 levels of our earlier compilation, is

log(lifetime)−log(C+T)=−0.798+0.00003311E,

where *E* is the value of the energy level in the traditional wavenumber units (cm^−1^).

A comparison of the 85 new determinations from other laboratories with our own revised values is given in table 1. The new measurements are given in column 2 under the heading “published”, and the values revised from those given by Corliss and Tech [1967] are given in column 3. The standard deviation from the mean of the ratios of the two values as given in column 4 is 30 percent. The literature references from which values obtained in the new direct measurements were taken are given in column 4.

The revised lifetimes are reported in table 2. The successive columns in this table contain the electron configurations, term designations, *J*-values, level values, lifetimes in nanoseconds given to two significant figures, and the number of downward transitions contributing to the lifetime determination. The configurations and term designations are taken from the compilation by Reader and Sugar [1975]. In some cases, the “less than” (<) symbol is prefixed to a lifetime value to indicate that the transition probability for an important downward transition from the level is lacking and could therefore not be used in the lifetime calculation. The value for the the lifetime in such cases must be regarded as an upper limit.

It should be noted that the information developed in this paper can also be used as a basis for improving the oscillator strengths, transition probabilities, and other quantities given in NBS Monograph 108 for 3288 lines of FeI. For each of the quantities listed in that monograph, a simple transformation expression can be written that is based on the correcting equation given above in this paper. For example, revised transition probabilities for the FeI lines listed in NBS Monograph 108 can be derived from the expression

$$\log\;A_{\text{rev}}=\log\;A_{108}+0.798+0.00003311\;E.$$

The relation between *f* and *A* is

$$g_{l}\;f=1.4992\times 10^{-16}\lambda^{2}g_{u}A$$

where *g_l_* is (2*J*+1) for the lower level, *g_u_* is (2*J*+1) for the upper level and λ is in Angstrom units. We also note that

$$\log\;g_{l}\;f=\log\;g_{u}A+2\;\log\;\lambda -15.824.$$

## Figures and Tables

**Figure 1:**
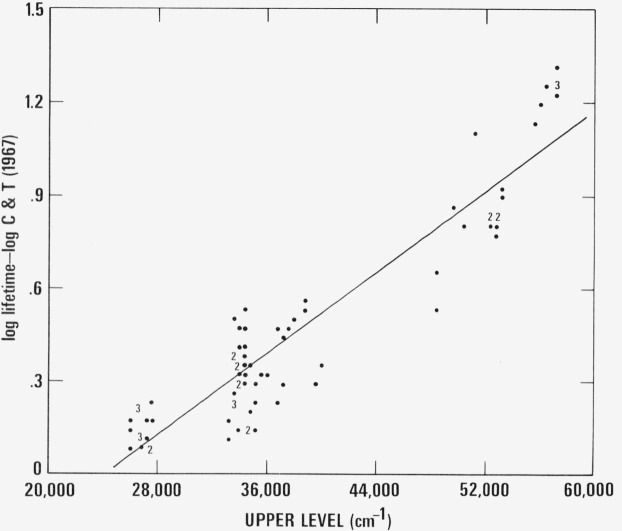
Logarithm of the ratio of directly measured lifetimes to those of Corliss and Tech [1967] plotted versus energy level value in *FeI*.

**Table 1:** Comparison of published lifetimes with present results

Level cm^−1^	Published ns	Present ns	Ratio	Reference
				
25900. 00	74. 6	68. 5	0. 92	Hilborn [1973]
25900. 00	89. 0	68. 5	. 77	Figger [1974]
26140. 19	87. 2	70. 7	. 81	Figger [1974]
26339. 71	94. 7	70. 0	. 74	Figger [1974]
26479. 39	91. 2	65. 2	. 71	Figger [1974]
26550. 50	88. 6	65. 5	. 74	Figger [1974]
26874. 56	59. 5	58. 5	. 98	Wagner [1969]
26874. 56	61. 5	58. 5	. 95	Klose [1971]
26874. 56	63. 2	58. 5	. 93	Hilborn [1973]
26874. 56	61. 9	58. 5	. 95	Erman [1974]
27166. 84	67. 1	64. 1	. 96	Klose [1971]
27166. 84	63. 3	64. 1	1. 01	Hilborn [1973]
27166. 84	62. 6	64. 1	1. 02	Erman [1974]
27394. 70	67. 3	58. 8	0. 87	Erman [1974]
27559. 60	66. 7	56. 2	. 84	Erman [1974]
27666. 36	68. 4	51. 4	. 75	Erman [1974]
33095. 96	5. 8	8. 7	1. 50	Assousa [1972]
33095. 96	6. 46	8. 7	1. 35	Hilborn [1973]
33507. 14	5. 8	6. 8	1. 17	Assousa [1972]
33507. 14	6. 1	6. 8	1. 11	Figger [1975]
33695. 42	11. 5	9. 7	0. 84	Klose [1971]
33695. 42	7. 9	9. 7	1. 23	Andersen [1971]
33695. 42	8. 4	9. 7	1. 15	Assousa [1972]
33695. 42	10. 4	9. 7	0. 93	Erman [1974]
33695. 42	8. 16	9. 7	1. 19	Figger [1974]
33801. 60	5. 8	8. 6	1. 48	Assousa [1972]
33946. 96	38. 6	30. 5	0. 79	Figger [1975]
34017. 13	6. 1	6. 5	1. 07	Figger [1975]
34039. 54	9. 1	8. 8	0. 97	Andersen [1971]
34039. 54	8. 4	8. 8	1. 05	Assousa [1972]
34039. 54	10. 7	8. 8	0. 82	Erman [1974]
34039. 54	8. 29	8. 8	1. 06	Figger [1974]
34121. 62	5. 9	5. 5	0. 93	Figger [1975]
34328. 78	8. 4	7. 8	. 93	Assousa [1972]
34328. 78	10. 9	7. 8	. 72	Erman [1974]
34328. 78	7. 92	7. 8	. 98	Figger [1974]
34362. 89	112. 1	101. 2	. 90	Figger [1975]
34547. 24	11. 0	7. 9	. 72	Erman [1974]
34547. 24	8. 00	7. 9	. 99	Figger [1974]
34555. 64	111. 5	70. 3	. 63	Figger [1975]
34692. 17	11. 0	7. 4	. 67	Erman [1974]
34692. 17	7. 86	7. 4	. 94	Figger [1974]
34782. 45	8. 3	13. 1	1. 58	Andersen [1971]
34844. 34	8. 8	14. 2	1. 61	Andersen [1971]
35257. 34	7. 6	12. 4	1. 63	Andersen [1971]
35379. 24	10. 6	12. 6	1. 19	Andersen [1971]
35379. 24	9. 6	12. 6	1. 31	Andersen [1971]
35767. 59	10. 2	11. 7	1. 15	Andersen [1971]
36079. 40	10. 3	12. 2	1. 18	Andersen [1971]
36686. 20	11. 5	10. 0	0. 87	Andersen [1971]
36767. 00	7. 2	10. 7	1. 49	Assousa [1972]
37157. 59	7. 2	9. 7	1. 35	Assousa [1972]
37162. 77	10. 7	10. 3	0. 96	Andersen [1971]
37521. 19	10. 2	9. 7	. 95	Andersen [1971]
38175. 38	9. 3	8. 5	. 91	Andersen [1971]
38678. 07	8. 9	7. 4	. 83	Andersen [1971]
38995. 76	7. 2	6. 5	. 90	Andersen [1971]
39625. 83	3. 2	5. 4	1 69	Assousa [1972]
39969. 88	3. 2	4. 5	1. 41	Assousa [1972]
48382. 63	13. 8	19. 5	1. 41	Whaling [1969]
48382. 63	11. 0	19. 5	1. 77	Andersen [1971]
49604. 45	7. 8	7. 5	. 96	Andersen [1971]
50522. 94	13. 0	14. 7	1. 13	Siomos [1975]
51373. 96	14. 0	8. 9	. 64	Andersen [1971]
52513. 59	8. 0	10. 8	1. 35	Whaling [1969]
52513. 59	8. 4	10. 8	1. 29	Andersen [1971]
52513. 59	8. 5	10. 8	1. 27	Andersen [1971]
52655. 04	9. 7	12. 9	1. 33	Whaling [1969]
52655. 04	9. 0	12. 9	1. 43	Andersen [1971]
52899. 06	8. 5	10. 9	1. 28	Andersen [1971]
52899. 06	8. 3	10. 9	1. 31	Andersen [1971]
53093. 60	9. 5	9. 7	1. 05	Whaling [1969]
53093. 60	8. 4	9. 7	1. 15	Andersen [1971]
55489. 91	8. 9	7. 2	. 81	Andersen [1971]
55905. 56	10. 2	7. 3	. 72	Andersen [1971]
56592. 76	9. 3	6. 2	. 67	Andersen [1971]
57027. 56	11. 4	8. 0	. 70	Whaling [1969]
57027. 56	12. 1	8. 0	. 66	Andersen [1971]
57070. 25	15.	8. 6	. 57	Whaling [1969]
57070. 25	13. 0	8. 6	. 66	Andersen [1971]
57104. 26	13. 4	9. 0	. 67	Andersen [1971]
*40257. 37	2. 6	9. 1	3. 50	Assousa [1972]
*40594. 47	2. 6	6. 4	2. 46	Assousa [1972]
*42911. 92	10. 2	25.	2. 45	Andersen [1971]
*51668. 22	12. 8	6. 5	. 51	Andersen [1971]

* Rejected outlying values.

**Table 2:** Radiative lifetimes for energy levels in neutral iron

Configuration	Term	*J*	Level cm^−1^	Lifetime ns	Number of transitions
					
3*d*^6^(^5^D)4*s*4*p*(^3^P^o^)	*z* ^7^D^o^	5	19350. 89	130000.	2
		4	19562. 46	52000.	2
		3	19757. 04	54000.	4
		2	19912. 51	65000.	2
		1	20019. 65	100000.	2
3*d*^6^(^5^D) 4*s*4*p* (^3^P^o^)	*z* ^7^F^o^	6	22650. 43	700000.	1
		5	22845. 88	11000.	2
		4	22996. 69	7700.	3
		3	23110. 95	9700.	2
		2	23192. 51	8900.	5
		1	23244. 85	15000.	4
3*d*^6^(^5^D)4*s*4*p*(^3^P^o^)	*z* ^7^P^o^	4	23711. 47	17000.	3
		3	24180. 88	30000.	4
		2	24506. 93	88000.	3
3*d*^6^(^5^D)4*s*4*p*(^3^P^o^)	*z* ^5^D^o^	4	25900. 00	69.	6
		3	26140. 19	71.	6
		2	26339. 71	70.	6
		1	26479. 39	65.	5
		0	26550. 49	66.	2
3*d*^6^(^5^D)4*s*4*p*(^3^P^o^)	*z* ^5^F^o^	5	26874. 56	59.	4
		4	27166. 84	64.	6
		3	27394. 70	59.	6
		2	27559. 60	56.	6
		1	27666. 36	51.	5
3*d*^6^(^5^D)4*s*4*p*(^3^P^o^)	*z* ^5^P^o^	3	29056. 34	34.	7
		2	29469. 03	36.	9
		1	29732. 75	30.	6
3*d*^6^(^5^D)4*s*4*p*(^3^P^o^)	*z* ^3^F^o^	4	31307. 27	410.	7
		3	31805. 10	450.	10
		2	32134. 01	680.	7
3*d*^6^(^5^D) 4*s*4*p*(^3^P^o^)	*z* ^3^D^o^	3	31322. 64	210.	9
		2	31686. 38	180.	7
		1	31937. 35	180.	6
3*d*^7^(^4^F)4*p*	*y* ^5^D^o^	4	33095. 96	8. 7	7
		3	33507. 14	6. 8	10
		2	33801. 59	8. 6	13
		1	34017. 13	6. 5	12
		0	34121. 62	5. 5	4
3*d*^7^(^4^F)4*p*	*y* ^5^F^o^	5	33695. 42	9. 7	5
		4	34039. 54	8. 8	10
		3	34328. 77	7. 8	9
		2	34547. 23	7. 9	8
		1	34692. 17	7. 4	6
3*d*^6^(^5^D)*s*4*p*(^3^P^o^)	*z* ^3^p^o^	2	33946. 96	30.	14
		1	34362. 89	100.	12
		0	34555. 64	70.	4
3*d*^7^(^4^F)4*p*	*z* ^5^G^o^	6	34843.98	14.	1
		5	34782. 45	13.	7
		4	35257. 34	12.	12
		3	35611. 65	11.	11
		2	35856. 42	11.	7
3*d*^7^(^4^F)4*p*	*z* ^3^G^o^	5	35379. 24	13.	7
		4	35767. 59	12.	12
		3	36079. 39	12.	13
3*d*^7^(^4^F)4*p*	*y* ^3^F^o^	4	36686. 20	10.	12
		3	37162. 77	10.	13
		2	37521. 19	9. 7	11
3*d*^6^(^5^D)4*s*4*p*(^1^P^o^)	*y* ^5^P^o^	3	36767. 00	11.	10
		2	37157. 59	9. 7	9
		1	37409. 57	8. 7	8
3*d*^7^(^2^F)4*s*	*d* ^3^F	2	36940. 60	<11000.	2
3*d*^7^(^4^F)4*p*	*y* ^3^D^o^	3	38175. 38	8. 5	16
		2	38678. 07	7. 4	14
		1	38995. 76	6. 5	16
3*d*^6^(^5^D)4*s*4*p*(^1^P^o^)	*x* ^5^D^o^	4	39625. 83	5. 4	7
		3	39969. 88	4. 5	13
		2	40231. 36	4. 2	13
		1	40404. 54	3. 5	9
		0	40491. 31	3. 6	3
3*d*^5^(^6^S)4*s*^2^4*p*	*y* ^7^P^o^	2	40052. 08	400.	6
		3	40207. 12	74.	7
		4	40421. 89	110.	4
3*d*^6^(^5^D)4*s*4*p*(^1^ P^o^)		5	40257. 37	9. 1	3
		4	40594. 47	6. 4	7
		3	40842. 13	5. 3	9
		2	41018. 06	4. 6	9
		1	41130. 62	3. 1	5
3*d*^6^(*a* ^3^P)4*s*4*p*(^3^P^o^)	*z* ^5^S^o^	2	40895. 02	29.	9
3*d*^6^(*a* ^3^P)4*s*4*p*(^3^P^o^)	*x* ^5^P^o^	3	42532. 76	32.	11
		2	42859. 83	19.	11
		1	43079. 05	46.	7
3*d*^6^(^3^H)4*s*4*p*(^3^P^o^)	*y* ^5^G^o^	6	42784. 39	23.	1
	*y* ^5^G^o^	5	42911. 92	25.	4
		4	43023. 00	30.	5
		3	43137. 51	21.	4
		2	43210. 04	19.	3
3*d*^6^(^5^D) 4*s*(^6^D)5*s*	*e* ^7^D	5	42815. 85	13.	7
		4	43163. 33	12.	10
		3	43434. 63	11.	10
		2	43633. 53	11.	8
		1	43763. 98	9. 5	6
3*d*^6^(^3^H)4*s*4*p*(^3^P^o^)	*z* ^5^H^o^	6	43321. 12	47.	1
		5	42991. 66	49.	5
		4	43108. 94	49.	5
		3	43325. 98	210.	5
3*d*^6^(a ^3^F)4*s*4*p*(^3^P^o^)	*w* ^5^D^o^	4	43499. 54	8. 9	8
		3	43922. 70	7. 5	10
		2	44183. 64	7. 6	14
		1	44411. 18	7. 1	13
		0	44458. 96	7. 7	3
3*d*^6^(a ^3^F) 4*s*4*p*(^3^P^o^)	^5^F^o^	5	44243. 67	33.	4
		4	44022. 55	57.	8
		3	44166. 24	41.	9
		2	44285. 48	35.	8
		1	44378. 42	35.	4
3*d*^6^(*a*^3^P)4*s*4*p*(^3^P^o^)	^5^D^o^	4	44415. 13	26.	6
		3	44551. 44	23.	6
		2	44664. 13	23.	10
		1	44760. 79	15.	5
		0	44826. 92	9. 4	2
3*d*^7^(^4^P)4*p*	*y* ^5^S^o^	2	44511. 86	5. 2	9
3*d*^6^(^5^D)4*s*(^6^D)5*s*	*e* ^5^D	4	44677. 01	22.	13
		3	45061. 33	22.	15
		2	45333. 88	19.	14
		1	45509. 15	19.	8
		0	45595. 08	17.	3
3*d*^6^(a ^3^P)4*s*4*p*(^3^P^o^)	*x* ^3^D^o^	3	45220. 74	15.	12
		2	45281. 89	14.	15
		1	45551. 83	12.	13
3*d*^6^(^3^H)4*s*4*p*(^3^P^o^)	*y* ^3^G^o^	5	45294. 86	40.	9
		4	45428. 46	42.	14
		3	45563. 03	95.	12
3*d*^6^(*a* ^3^F)4*s*4*p*(^3^P^o^)	*x* ^5^G^o^	6	45608. 35	46.	1
		5	45726. 18	31.	5
		4	45833. 24	20.	7
		3	45913. 53	23.	7
		2	45964. 98	20.	4
3*d*^6^(^3^H)4*s*4*p*(^3^P^o^)	*z* ^3^I^o^	7	45978. 04	120.	1
		6	46026. 98	150.	3
		5	46135. 92	180.	3
3*d*^7^(^4^P)4*p*	*w* ^5^P^o^	3	46137. 14	1. 5	4
		2	46313. 61	1. 6	6
		1	46410. 44	1. 4	8
3*d*^6^(*a* ^3^P)4*s*4*p*(^3^P^o^)	*z* ^3^S^o^	1	46600. 88	2. 8	11
3*d*^7^(^4^P)4*p*	*y* ^3^P^o^	0	46672. 57	16.	5
		1	46901. 89	6. 5	13
		2	46727. 14	8. 3	10
3*d*^6^(*a* ^3^F)4*s*4*p*(^3^P^o^)	^3^F^o^	4	46720. 85	8. 9	15
		3	47092. 78	10.	17
		2	47197. 07	14.	14
3*d*^7^(^4^P)4*p*	^5^D^o^	4	46889. 21	9. 3	14
		3	47017. 24	7. 3	15
		2	47136. 14	6. 5	16
		1	47177. 25	5. 6	12
		0	47171. 52	3. 7	4
3*d*^7^(^4^P)4*p*	^3^D^o^	3	46745. 03	2. 1	17
		2	46888. 58	3. 2	14
		1	47272. 09	9. 4	11
3*d*^6^(^3^H)4*s*4*p*(^3^P^o^)	*z* ^3^H^o^	6	46982. 38	30	7
		5	47008. 43	23.	14
		4	47106. 54	17.	16
3*d*^7^(^4^F)5*s*	*e* ^5^F	5	47005. 51	24.	9
		4	47377. 97	24.	17
		3	47755. 54	22.	19
		2	48036. 67	22.	16
		1	48221. 32	18.	12
3*d*^6^(^3^G)4*s*4*p*(^3^P^o^)	*w* ^5^G^o^	6	47363. 39	35.	5
		5	47420. 23	19.	7
		4	47590. 07	32.	10
		3	47693. 29	<38.	8
		2	47831. 20	25.	7
*3d*^6^(*a* ^3^P)4*s*4*p*(^3^P^o^)	^1^D^o^	2	47419. 72	20.	13
3*d*^7^(^2^G)4*p*	*z* ^1^G^o^	4	47452. 77	62.	13
3*d*^7^(^4^P)4*p*	*y* ^3^S^o^	1	47555. 63	9. 7	13
3*d*^6^(^3^G)4*s*4*p*(^3^P^o^)	*v* ^5^F^o^	5	47606. 10	25.	6
		4	47930. 04	13.	13
		3	48122. 97	14.	11
		2	48238. 90	9. 4	17
		1	48350. 62	9. 5	7
3*d*^6^(^3^G)4*s*4*p*(^3^P^o^)	^5^H^o^	3	47834. 26	41.	12
		4	47812. 18	41.	15
		5	47834. 62	35.	10
3*d*^7^(^4^F)5*s*	*e* ^3^F	4	47960. 97	29.	19
		3	48531. 90	26.	20
		2	48928. 42	25.	9
3*d^5^*(^6^S)4*s*^2^4*p*	*v* ^5^P^o^	3	47966. 63	1. 4	6
		2	48163. 49	4. 3	13
		1	48289. 89	5. 4	9
3*d*^6^(*a* ^3^F)4*s*4*p*(^3^P^o^)	*w* ^3^G^o^	5	48231. 33	120.	9
		4	48361. 92	100.	8
		3	48475. 74	39.	9
3*d*^6^(*a* ^3^P)4*s*4*p*(^3^P^o^)	*x* ^3^P^o^	2	48304. 71	8. 4	19
		1	48516. 15	9. 4	14
		0	48460. 12	9. 0	7
3*d*^7^(^2^G)4*p*	*z* ^1^H^o^	5	48382. 63	20.	12
3*d*^6^(^3^H)4*s*4*p*(^3^P^o^)	*y* ^1^G^o^	4	48702. 57	17.	13
	2^o^	2	49052. 93	48.	2
3*d*^7^(^2^ G)4*p*	*w* ^3^F^o^	4	49108. 94	10.	11
		3	49242. 95	9. 9	14
		2	49433. 18	8. 7	11
3*d*^6^(*a* ^3^F)4*s*4*p*(^3^P^o^)	*v* ^3^D^o^	3	49135. 08	12.	11
		2	49242. 68	11.	12
		1	49297. 66	11.	13
3*d*^6^(*a* ^3^F)4*s*4*p*(^3^P^o^)	^1^F^o^	3	49227. 16	210.	1
3*d*^7^ (^2^G)4*p*	*y* ^3^H^o^	6	49434. 20	10.	7
		5	49604. 45	7. 5	9
		4	49727. 06	5. 6	12
3*d*^7^(^2^G)4*p*	*v* ^3^G^o^	5	49460. 92	7. 3	12
		4	49627. 92	7. 0	16
		3	49850. 61	6. 1	11
	*z* ^1^D^o^	2	49477. 10	65.	10
3*d*^6^(^5^D)4*s*(^6^D)5*p*	*x* ^7^P^o^	3	49804. 90	55.	1
3*d*^7^(^2^P)4*p*	*w* ^3^P^o^	0	49951. 36	7. 8	6
		1	50043. 25	9. 3	13
		2	50186. 87	11.	11
3*d*^6^(^5^D)4*s* (^6^D)4*d*	*e* ^7^F	6	50342. 18	4. 7	3
		5	50833. 48	4. 6	9
		4	51192. 32	4. 3	11
		3	51148. 87	5. 0	12
		2	51331. 09	6. 6	10
		1	51208. 04	3. 9	6
3*d*^6^(^5^D)4*s* (^6^D)4*d*	*f* ^7^D	5	50377. 92	5. 3	4
		4	50808. 05	4. 1	13
		3	50861. 85	4. 5	7
		2	50998. 69	5. 7	13
		1	51048. 10	5. 1	12
3*d*^6^(^5^D)4*s*(^6^D)4*d*	*f* ^5^D	4	50423. 18	5. 9	14
		3	50534. 43	5. 6	17
		2	50698. 67	8. 9	15
		1	50880. 15	9. 6	10
		0	50981. 02	7. 8	6
3*d*^6^(^5^D)4*s*(^6^D)4*d*	*e* ^7^P	4	50475. 32	6. 4	8
		3	50611. 30	8. 8	13
		2	50861. 32	15.	9
3*d*^6^(^5^D)4*s*(^6^D))4*d*	*e* ^5^G	6	50522. 94	15.	6
		5	50703. 91	9. 9	10
		4	50979. 63	14.	10
		3	51219. 06	7. 3	11
		2	51370. 18	9. 1	12
3*d*^7^(^2^G)4*p*	*z* ^1^F^o^	3	50586. 89	19.	11
*3d*^6^(*a* ^3^F)4*s*4*p*(^3^P^o^)	*x* ^1^G^o^	4	50614. 02	29.	7
3*d*^6^(^5^D)4*s*(^6^D)4*d*	*e* ^7^G	7	50651. 76	5. 6	1
		6	50967. 87	7. 1	4
		5	51228. 59	7. 0	9
		4	51334. 94	6. 3	13
		3	51460. 53	4. 6	8
		2	51539. 77	4. 7	6
		1	51566. 86	4. 9	4
3*d*^6^(^5^D)4*s*(^6^D)5*p*	*u* ^5^F^o^	5	51016. 72	58.	2
		4	51381. 48	49.	3
		3	51619. 14	100.	3
		2	51827. 59	18.	4
3*d*^6^(^3^G)4*s*4*p*(^3^P^o^)	*x* ^3^H^o^	6	51023. 19	54.	7
		5	51068. 77	49.	12
		4	51409. 18	22.	11
3*d*^6^(^5^D)4*s*(^6^D)5*p*	*t* ^5^D^o^	4	51076. 68	<49.	2
		3	51361. 46	22.	8
		2	51630. 07	22.	6
		1	51836. 87	66.	1
		0	51941. 76	42.	1
3*d*^6^(^5^D)4*s*(^6^D)4*d*	*f* ^5^F	5	51103. 24	10.	9
		4	51461. 71	9. 0	11
		3	51604. 15	17.	13
		2	51705. 05	17.	12
		1	51754. 53	28.	8
3*d*^6^(^5^D)4*s* (^6^D)4*d*	*e*^5^S	2	51148. 89	8. 3	8
3*d*^7^(*a*^2^D)4*p*	*v* ^3^F^o^	2	51201. 33	19.	11
		3	51365. 30	28.	9
		4	51304. 65	16.	13
3*d*^6^(^5^D)4*s*(^4^D)5*s*	*e* ^3^D	3	51294. 26	20.	19
		2	51739. 96	17.	19
		1	52039. 94	22.	10
3*d*^6^(^5^D)4*s*(^4^D)5*s*	*g* ^5^D	4	51350. 50	12.	19
		3	51770. 58	13.	23
		2	52049. 82	16.	23
		1	52214. 33	13.	19
		0	52257. 33	13.	6
3*d*^6^(^3^G)4*s*4*p*(^3^P^o^)	*u* ^3^G^o^	5	51373. 96	8. 9	11
		4	51668. 22	6. 5	18
		3	51825. 80	9. 1	10
	5^o^	3	51435. 90	83.	3
3*d*^6^(^5^D)4*s*(^6^D)4*d*	*e* ^7^S	3	51570. 16	4. 1	11
3*d*^6^(^3^H)4*s*4*p*(^3^P^o^)	^1^H^o^	5	51630. 23	28.	8
3*d*^6^(^5^D)4*s*(^6^D)5*p*	*u* ^3^P^o^	3	51691. 98	580.	1
3*d*^6^(*a* ^3^F)4*s*4*p*(^3^P^o^)	*y* ^1^D^o^	2	51708. 33	11.	11
	7^o^	2	51756. 16	57.	3
3*d*^7^(^2^P)4*p*	*x* ^1^D^o^	2	51762. 12	42.	6
3*d*^6^(^5^D)4*s* (^6^D)4*d*	*e* ^5^P	3	51837. 28	20.	13
		2	52067. 45	22.	15
		1	52019. 71	23.	8
3*d*^7^(^2^P)4*p*	*u* ^3^D^o^	3	51969. 14	6. 8	12
		2	52296. 96	4. 0	11
		1	52512. 46	3. 3	14
3*d*^7^(^2^P)4*p*	^1^P^o^	1	52180. 82	17.	8
3*d*^7^(*a* ^2^D)4*p*	^3^D^o^	3	52213. 29	9. 3	10
		2	52682. 93	10.	11
		1	53229. 94	5. 2	11
3*d*^7^(^2^H)4*p*	*w* ^3^H^o^	6	52431. 47	8. 3	5
		5	52613. 08	7. 4	6
		4	52768. 78	9. 4	7
3*d*^7^(^2^H)4*p*	*y* ^3^I^o^	7	52655. 04	13.	2
		6	52513. 59	11.	7
		5	52899. 06	11.	6
3*d^7^*(*a* ^2^D)4*p*	^3^p^o^	1	52857. 84	4. 1	11
		2	52916. 33	5. 5	9
	*s* ^3^D^o^	3	52953. 68	36.	4
3*d*^7^(^4^F)4*d*	*g* ^5^F	5	53061. 28	27.	6
		4	53393. 71	35.	11
		3	53830. 96	41.	11
		2	54257. 52	73.	6
		1	54386. 16	19.	10
3*d*^7^(^2^H)4*p*	*z* ^1^I^o^	6	53093. 60	9. 7	4
3*d*^7^(^4^F)4*d*	*h* ^5^D	4	53155. 13	40.	6
		3	53545. 88	25.	11
		2	53966. 72	27.	8
		1	54132. 48	<52.	6
	*f* ^5^P	3	53160 53	<44.	4
		2	53568. 72	20.	12
		1	53925. 26	25.	11
3*d*^7^(^4^F)4*d*	*f* ^5^G	6	53169. 21	38.	5
		5	53281. 73	17.	6
		4	53769. 02	28.	12
		3	54161. 18	20.	12
		2	54375. 72	35.	5
3*d*^7^(^4^F)4*d*	*e* ^5^H	7	53275. 20	40.	1
		6	53353. 02	35.	2
		5	53874. 30	31.	3
		4	54237. 20	36.	3
		3	54491. 08	38.	1
3*d*^6^(^3^D)4*s*4*p*(^3^P^o^)	^5^F^o^	2	53275. 27	73.	2
		5	54013. 78	17.	5
	9^o^	4	53328. 87	31.	5
		3	53388. 68	92.	1
3*d*^7^(a ^2^D)4*p*	*y* ^1^F^o^	3	53661. 13	18.	9
3*d*^6^(^3^G)4*s*4*p*(^3^P^o^)	*y* ^1^H^o^	5	53722. 44	16.	2
3*d*^7^(^4^F)4*d*	*e* ^3^G	5	53739. 49	22.	12
		4	54066. 57	27.	9
		3	54379. 44	37.	3
3*d*^7^ (^4^F)4*d*	*f* ^3^D	3	53747. 55	25.	12
		2	54066. 82	22.	14
		1	54449. 33	10.	14
3*d*^6^(^3^G)4*s*4*p*(^3^P^o^)	*x* ^3^F^o^	3	53763. 28	15.	10
3*d*^6^(^5^D)4*s*(^6^D)6*s*	*g* ^7^D	5	53800. 90	38.	4
		4	54124. 62	22.	6
		2	54611. 72	<41.	4
		1	54747. 74	34.	3
3*d*^7^ (^2^P)4*p*	^3^S^o^	1	53808. 37	4. 5	12
3*d*^7^ (^4^F)4*d*	*e* ^3^H	6	53840. 68	34.	2
		5	54266. 76	200.	1
		4	54555. 45	30.	4
3*d*^6^(^3^D)4*s*4*p*(^3^P^o^)	^5^D^o^	3	53891. 54	11.	9
		4	54301. 36	20.	9
3*d*^7^(^2^H)4*p*	*t* ^3^G^o^	5	53983. 30	6. 5	8
		4	54237. 46	7. 8	11
		3	54600. 35	6. 8	13
3*d*^6^(^3^D)4*s*4*p*(^3^P^o^)	^5^p^o^	3	54004. 82	95.	7
		2	54112. 30	11.	3
		1	54271. 11	20.	6
3*d*^7^(^4^F)4*d*	*f* ^3^F	4	54683. 39	37.	9
		3	55124. 97	27.	9
		2	55378. 84	23.	6
3*d*^6^(^3^ G)4*s*4*p*(^3^P^o^)	*w* ^1^G^o^	4	54810. 82	31.	4
3*d*^7^(^4^F)4*d*	*e* ^3^P	2	54879. 72	40.	5
		1	55376. 12	27.	5
		0	55726. 54	12.	2
3*d*^6^(a ^1^G)4*s*4*p*(^3^P^o^)	*v* ^3^H^o^	4	55446. 06	9. 6	6
		5	55429. 89	8. 1	9
		6	55489. 81	7. 2	5
3*d*^7^(^2^H)4*p*	*x* ^1^H^o^	5	55525. 58	22.	5
3*d*^7^(*a* ^2^D)4*p*	*w* ^1^D^o^	2	55754. 29	1. 9	8
3*d*^6^(^3^G) 4*s*4*p*(^3^*P*^o^)	*w* ^1^F^o^	3	55790. 72	8. 3	8
3*d*^6^ (*a* ^1^G)4*s*4*p*(^3^P^o^)	*s* ^3^G^o^	5	55907. 22	8. 4	7
		4	55905. 56	7. 3	9
		3	56097. 85	15.	3
3*d*^6^(^1^I)4*s*4*p*(^3^P^o^)	*u* ^3^H^o^	6	56334. 01	10.	4
		5	56382. 69	5. 3	7
		4	56423. 33	5. 4	9
	1	5	56428. 06	11.	4
	2	4	56452. 04	15.	5
3*d*^6^(*a* ^1^G)4*s*4*p*(^3^P^o^)	*u* ^3^F^o^	4	56592. 76	6. 2	6
		3	56783. 33	6. 4	6
		2	56858. 65	5. 3	
	3	4	56842. 70	12.	5
3*d*^7^(^2^H)4*p*	*v* ^1^G^o^	4	56951. 27	5. 1	5
3*d*^6^(^1^I)4*s*4*p*(P^o^)	*x* ^3^I^o^	7	57027. 56	8. 0	2
		6	57070. 25	8. 6	3
		5	57104. 26	9. 0	3
3*d*^6^(^3^D)4*s*4*p*(^3^P^o^)	*t* ^3^F^o^	4	57550. 09	6. 5	5
		3	57641. 06	16.	8
		2	57708. 76	5. 7	5
3*d*^6^(^5^D)4*s*(^4^D)4*d*	*i* ^5^*D*	4	57697. 59	7. 6	7
		3	57813. 97	7. 3	8
		2	57974. 16	6. 5	9
3*d*^6^(^6^D) 4*s*(^6^D)7*s*	*h* ^7^D	5	57897. 17	36.	2
3*d*^6^(^5^D)4*s*(^4^D)4*d*	*g* ^5^*G*	6	58001. 88	6. 6	3
		5	58271. 50	24.	4
		4	58520. 18	30.	5
		3	58710. 09	<120.	2
		2	58824. 81	30.	3
	4	2	58213. 17	9. 6	7
	*r* ^3^G^o^	5	59926. 62	5. 7	1
		4	60172. 06	2. 0	3
		3	60364. 76	1. 4	2
	*t* ^3^H^o^	6	60365. 70	2. 7	4
		5	60549. 18	2. 6	4
		4	60757. 68	2. 9	5
	*q* ^3^G^o^	3	60806. 72	5. 3	4
		3	53357. 53	36.	7
		4	53610. 44	73.	3
		3	53733. 51	81.	6
		2	53749. 39	28.	6
		3	53784. 74	64.	3
		4	53881. 91	69.	4
		3	54289. 09	46.	7
		3	54357. 40	23.	7
		3	57565. 35	32.	4
		3	60563. 61	15.	5
		2	62081. 27	21.	3
Fe II(^6^D_9/2_)	Limit		63480		

## Footnotes

1 Names and dates in brackets indicate the literature references at the end of the paper.
